# Computing Drug-Drug Similarity from Patient-Centric Data

**DOI:** 10.3390/bioengineering10020182

**Published:** 2023-02-01

**Authors:** Yousef Asiri

**Affiliations:** Department of Computer Science, Najran University, Najran 61441, Saudi Arabia; yasiri@nu.edu.sa

**Keywords:** text-based analysis, natural language processing, drug-drug similarity, social media, online healthcare communities

## Abstract

In modern biology and medicine, drug-drug similarity is a major task with various applications in pharmaceutical drug development. Various direct and indirect sources of evidence obtained from drug-centric data such as side effects, drug interactions, biological targets, and chemical structures are used in the current methods to measure the level of drug-drug similarity. This paper proposes a computational method to measure drug-drug similarity using a novel source of evidence that is obtained from patient-centric data. More specifically, patients’ narration of their thoughts, opinions, and experience with drugs in social media are explored as a potential source to compute drug-drug similarity. Online healthcare communities were used to extract a dataset of patients’ reviews on anti-epileptic drugs. The collected dataset is preprocessed through Natural Language Processing (NLP) techniques and four text similarity methods are applied to measure the similarities among them. The obtained similarities are then used to generate drug-drug similarity-based ranking matrices which are analyzed through Pearson correlation, to answer questions related to the overall drug-drug similarity and the accuracy of the four similarity measures. To evaluate the obtained drug-drug similarities, they are compared with the corresponding ground-truth similarities obtained from DrugSimDB, a well-known drug-drug similarity tool that is based on drug-centric data. The results provide evidence on the feasibility of patient-centric data from social media as a novel source for computing drug-drug similarity.

## 1. Introduction

Drug-Drug Similarity (DDS) has received a lot of attention in recent years from biomedical researchers as a result of its usefulness in treating medical issues. It aims to find drugs with similar traits to a given drug resting on a general assumption that similar drugs share similar characteristics such as chemical structure [1], gene expression profiles [2], side effect profiles [3], and biological target [4]. In pharmaceutical drug development, in particular, DDS has successfully applied for drug repositioning [4,5], drug-drug interaction prediction [6,7], drug target identification [3], and drug side-effects prediction [7]. Each of these applications is driven by an application-specific hypothesis. A drug repositioning application, for instance, is motivated by the idea that if two different medications, D1 and D2, have comparable modes of action and properties, and D1 is utilized to treat a certain condition S, then D2 has the potential to be a choice to treat condition S. In drug-drug interaction prediction applications, the hypothesis is if drug D1 interacts with drug D2, and drug D3 is similar to D1, then D3 should also interact with D2 (the argument also follows if D1 is replaced with D2). The application of drug side-effect prediction is based on the hypothesis that if drug D1 is similar to drug D2 and drug D1 is known to cause a certain side effect, then drug D2 should also cause the same side effect.

The computation of DDS is essentially based on applying data similarity methods to drug-centric of different types. In doing so, these methods utilize different data similarity measures, which vary according to the type of data. Nonetheless, these measures can be divided into three broad categories [8]. The first category measures DDS using different features of drug and targets such as Anatomical Therapeutic Chemical (ATC) codes, molecular structure of drugs, sequences, and gene ontology of targets. The second category measures DDS using relationships such as drug-drug interaction, associations of drug–disease, and associations of drug–target. Finally, the third category integrates multi-information from multiple data sources to measure DDS. It should be noted that the computation of DDS has been made possible by recent developments in high-throughput biology, which have generated enormous quantities of data focused on drugs. The pharmacological side effects, gene ontologies, chemical structures, targets, and ATC codes are some examples of the data that are curated in biomedical databases such as DrugBank, CHEMBL, PubChem, SIDER, and KEGG. The chemical structure of a drug is a three-dimensional description of drug structure using strings of characters. The World Health Organization has adopted the ATC classification system, which divides each level of drug classification into a number of classes based on each level’s characteristics and therapeutic effects. Proteins and nucleic acids are examples of biological macromolecules that can be affected by a drug to carry out its pharmacodynamic actions in the body. Furthermore, gene ontology of drug targets is a representation of the ways in which gene products function in the biological realms. It is a helpful data source for biomedical research that is employed in the computer analysis of large-scale genetics and biological experiments. Finally, the drug side effects reported undesirable effects that may occur at standard doses should be considered throughout the drug targeting procedure [8].

Social media has recently become a valuable data source for healthcare informatics [9]. The emergence of Web 2.0 and Health 2.0 has made it possible for patients to share their social media experiences with illnesses, treatments, drug names, physicians, and therapists. Consequently, a massive amount of health information becomes available, representing potentially valuable, yet largely unexploited data sources that could be leveraged for drug knowledge discovery [10]. In this regard, the enormous amount of healthcare text generated from social media sites such as Google, Twitter, and YouTube has been used to tackle a number of medical issues such as detection of psychopathic class [11,12], classification of depression [13], identification of diseases [14], and detection of adverse drug reactions [15].

On this basis, this paper argues that social media data in the form of patient narration of their thoughts, opinions, and experience with drugs represent a potential source of drug-centric data that could be utilized for measuring DDS. It is based on a new drug-drug similarity hypothesis that states similar drugs should share similar aspects of patients’ experience. As the patients experience in social media is expressed in textual form, the problem of DDS is formulated as text similarity problems to which text similarity approaches can be applied. In Natural Language Processing (NLP), text similarity plays an important role in many tasks such as automatic translation, information retrieval, intelligent responses, and machine matching for dialogues and documents [16]. Over the past three decades, various semantic similarity techniques have been proposed and used in different contexts. Following this idea, each drug will be modeled as a document which contains all posts written about it. In this space of documents, text similarity can be applied to measure the similarity among them. The idea of utilizing patient-centric data in social media as a data source for measuring DDS is distinguished from the drug-centric data sources in three ways. First, unlike the drug-centric data which are stored in structured databases, the patient-centric data are unstructured. Second, the patient-centric data are produced by patients, who typically write simply and plainly without using professional medical terms when expressing their experiences on medical concerns, as opposed to the drug-centric data, for which a professional medical language is employed [17]. Because of this, the DDS method used with social media is different from the method used with more conventional drug-centric data sources in that it mainly relies on NLP techniques to extract pertinent information from social media. Third, the drug-centric data represent professionals’ perspectives, whereas the patients-centric data reflect patients’ thoughts and opinions. It is worth mentioning that this research has two-fold contributions: it introduces a new domain of applications where social media can be utilized and it adds a new data source that is worth exploration.

Finally, from a practical perspective, it is expected that the outcomes of this research would have a significant impact on the practical applications of DDS in drug discovery and development such as drug repositioning, drug side-effect prediction, and drug-drug interaction prediction. This is due to the fact that pharmaceutical corporations now place a high priority on incorporating patient perspectives into drug discovery and development [10]. Since the currently used methods for computing DDS depend only on drug-related data, this research would meet the requirement of incorporation of patients’ perspective in DDS and its practical applications. For example, in the application of DDS for the prediction of drug-drug interaction, the proposed patient’s centric data can be integrated with the traditional drug-centric data for a more robust computation of DDS which consequently improves the prediction of drug-drug interaction.

## 2. Background

The computation of DDS measures the similarity between drugs from drug-centric data sources. Usually, the resultant similarities are used as input to a target application. In this section, the previous DDS works which rely on computing DDS from various drug-centric data sources, regardless of the target application, are reviewed in the following dimensions: source of drug-centric data, similarity measures, target applications.

In the first dimension, source of drug-centric data, the previous DDS works utilizes different drug-centric data sources such as chemical structure [1,18], gene expression profiles [2], protein targets [19,20], side-effect profile [3,21], and clinical information [22]. It should be mentioned that in addition to the previous DDS works that utilizes a single drug-related data source, many DDS works utilize multiple drug-centric data sources to compensate for missing data across individual data sources and provide a multi-view aspects for forecasting related medications. Thus, a new insights into the target application [23,24]. In some of these works, drug characteristic is regarded as the combination of many drug similarities. In [23], GIPAE, for instance, combines chemical structure similarity from SMILES data as measured by Chemistry Development Kit (CDK), and association similarity from drug–disease association profiles as measured by the GIP kernel to represent drug features. Using the combined similarities as drug feature, the computation of DDS has improved drug–disease association prediction. Many works have proposed various integration approaches to leverage multimodal data and fuse similarities more effectively, but some of the earlier works integrated multiple similarities to yield multiple similarity matrices. These approaches of integration can be categorized as either linear integration or nonlinear integration [8].

As for the employed similarity measure, these measures can be a general similarity measure specific to drug-related data. While the general similarity measures such as Jaccard Coefficient, Euclidean distance, Cosine similarity are domain-independent measures that can be used in any domain, the drug-centric data-specific similarity measures such as CDK [25], SIMCOMP [26], normalized Smith–Waterman algorithm [27], GOSemSim [28] are developed specifically to measure the drug-drug similarity. The CDK is a library of structural chemoinformatics and bioinformatics developed in Java programming language and to perform many molecular informatics tasks such as two- and three-dimensional representations of chemical structures, structure diagram generation, SMILES parsing and generation, I/O routines, isomorphism checking, ring searches, etc. SIMCOMP is a method that compares chemical structures using graphs, represented as a two-dimensional graph with vertices for atoms and edges for covalent bonds. This approach counts the number of similar atoms in comparison between the corresponding graphs of two chemical compounds to determine how similar they are. By using a local sequence alignment to compare segments of all practical lengths, the Smith–Waterman method analyzes two protein canonical sequences of pharmaceutical targets. Next, the similarity between the similar parts is calculated.

Based on the target applications in which the computation of DDS is utilized, the following domains of applications can be identified:

- Drug repositioning: to discover new uses for existing drugs according to the similar compounds of drugs that are expected to interact with similar signs. Because it is a very effective strategy with low risk and cost, drug repositioning for DDS has many successful applications in drug development [29].
- Drug side effect prediction: to predict unexpected side effects of a drug based on computing ligand similarity and protein interactions. Knowing affected biological pathways and binding partners of a given drug is important for predicting both its efficacy and side effects [30]. The similarity-based drug side-effect prediction is an effective strategy, because the currently used laboratory assay method for evaluating potential adverse drug effects is a time-consuming method with high cost.
- Drug-Drug Interaction: the interaction between two drugs taken concomitantly occurs when the action of one of them intervenes with the activity of the other. The discovery of the interaction between drugs is of significant benefit for guidance of clinical medications, because it could lead to adverse drug reactions or complicate disease treatments on patients. The similarity-based method is one of the successful methods to identify drug-drug interactions [8].
- Drug–disease associations: the discovery of yet-unknown links between drugs and diseases has gained significant attention. In this regard, the similarity-based methods play an important role in complementing or guiding costly and exhausting wet experiments. In addition, the prediction of novel associations between drug and disease can be done utilizing the previously known drug–disease associations and the features of drug and disease as well [31].
- Drug–target interaction prediction: to forecast a possible relationship between a medicine and a target. It is a necessary stage for tasks such as drug discovery and repositioning. In the database, similar medications and targets can be found using similarity-based algorithms and based on the known interactions between these drugs and targets, the interaction can be predicted [32].
- Personalized medicine: to fit a treatment according to the characteristics of each patient. It requires a grouping of patients into subgroups with predictable response to a specific treatment. In this regards, the exploratory and predictive analysis provided by the similarity-based methods supports clinical decision-making, which is a key step in personalized medicine [1].

In all the previous works, the drug-centric data are a key factor for computing DDS. Moreover, in all of the previous works, the source of drug-related data represents professional perspective of the drugs. From the perspective of modern-day business dynamics, integrating patients’ perspectives into drug discovery and development is a critical issue. Furthermore, in the recent years, patient-perceived benefits are receiving increasing attention by the pharmaceutical regulatory authorities when decisions such as drug-approval, pricing, and reimbursement are made. The analysis of existing research shows that people with major diseases and disabilities have a propensity to use social media to seek self-help by sharing their experiences with their conditions [10,33]. Interestingly, the examination of patient posts on these social media platforms could be used to glean insightful information that opens the door for patient-centered drug development.

On this basis, this work intends to incorporate the patient’s perspective in the computation of DDS by considering his/her experience and opinions on the drug as a new source of drug-centric data for computing DDS. Unlike, the conventional drug-related data sources, in which data are curated in a structural form, the patient’s experience about the drugs in social media is unstructured and, therefore, the computation of DDS requires employing text similarity.

Text similarity is a ubiquitous notion within the natural language processing (NLP) community. It is utilized in a wide range of tasks such as question answering [34], automatic essay grading [35], or paraphrase recognition [36]. The text similarity methods can be divided into three broad categories [37]: string-based similarity method, corpus-based similarity method, and knowledge-based similarity method. String metric similarity or dissimilarity (distance) between two strings is used in the string-based similarity approach (also known as lexical-based similarity). The corpus-based similarity method (semantic-based similarity) calculates how similar two words are by using data from huge corpora. On the other hand, the knowledge-based similarity method calculates the degree of similarity between words using data from semantic networks such as WordNet, a sizable lexical database of English words created specifically for this purpose. Semantic relatedness measurements and semantic similarity measures are additional categories for knowledge-based similarity metrics. While the semantic similarity measures evaluate the similarity between concept based on their likeness, semantic relatedness measures employ a more general notion of relatedness that is not tied specifically to the form or shape of the concept.

## 3. Materials and Methods

**Figure 1:**
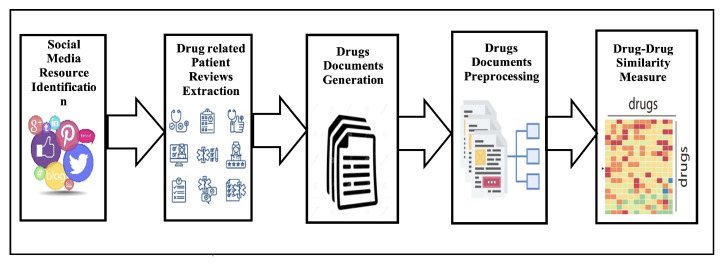
Drug–Drug similarity computation from social media.

The task of computing drug-drug similarity can be viewed as a use case of the general task of drug knowledge discovery that is concerned with extracting insights from available data. The five key stages of the standard approach for extracting drug-related datasets from social media [10] are: (1) resource selection, (2) dataset extraction, (3) data preparation, (4) data analysis, and (5) overall evaluation. The main elements of the process are frequently preserved, even though the specifics of each step may change depending on the final application. Figure 1 depicts the specific use case of the general drug knowledge discovery methodology that is concerned with computing drug-drug similarity from social media platforms.

In the first stage of this process, the social media resource of patient’s reviews should be identified. In general, the patients’ reviews of drugs can be drawn from social media platforms, which are divided into general platforms and specialized healthcare platforms. Facebook, Twitter, Instagram, and Reddit represent general social media platforms. The specialized healthcare social media platforms are divided into three types: generic health-centered platforms, drugs-focused sharing platforms, and disease-specific platforms. While the generic health-centered platforms, such as Patients-LikeMe, DailyStrength, MedHelp, WebMD, and CureTogether, permit patients to communicate their experiences on health-related issues, the drug-focused sharing platforms, such as Askapatient and Medications.com, permit patients to discuss and share their experiences on medications. On the other hand, disease-specific platforms focus on particular diseases, e.g., the TalkStroke forum [15].

After the identification of the social media data source, the second step is to extract patient-centric data from the identified social media platforms. For this purpose, two types of processes can be utilized: focused crawling and Web scraping. Focused crawling refers to automatically collecting websites that satisfy given criteria, e.g., all websites on Alzheimer’s disease or all websites on public health topics from a particular domain. In this process, the crawling algorithm should implement hyperlink analysis and prioritization processes to exclude many irrelevant sites. On the other hand, the Web scraping process refers to automated and systematic extraction of specific content of interest from given webpages. The decision of which process can be utilized is made based on the type of identified social media platform from which patients’ reviews are extracted. More specifically, to extract patients’ reviews from generic health-centered platforms, specific application programming interfaces can be used; however, an adapted web crawler to collect web pages and web scraper is usually used to obtain the patients’ reviews from specialized healthcare social networks [38].

The third step in the methodology of computing DDS is to generate drug documents. In this process, a single document that contains all collected patients’ reviews must be generated for each drug. The document of a given drug is generated by aggregating all collected patients reviews on that drug into a single document. The results of this step is a set of documents equal to the number of drugs under consideration.

The fourth step is the preprocessing of drug documents using NLP techniques to facilitate insightful analysis by reducing noise and structuring the text of drug documents. Data preparation and data reduction can often be used to execute the preprocessing in two steps. Data cleaning, standardization, and transformation are steps in the data preparation process. While data cleaning aims to ensure that complete and concise data are available and free from duplicates by applying appropriate techniques such as word removal, and repost removal, the aim in the data standardization is to ensure the data are expressed in unified medical form by identifying all imprecise medical terms and concepts occurrences in social-media posts and replacing them with appropriate ones. In data transformation, the data are instead transformed into a format that may be used for analysis. In the data reduction step, the dimensionality of the data is decreased using techniques including feature selection, transformation, and instance selection. When the data dimensionality is enormous, as in the case of text in drug documents, feature transformation, which seeks to condense original features into a limited set, is a critical procedure. On the other hand, by removing posts that are not relevant, for example, instance selection seeks to reduce the size of the data without sacrificing important information. Finally, feature selection is carried out by removing as many redundant and unnecessary features from the data as is practical.

After obtaining the drug documents in vector space model format, it is possible to compute the DDS by using similarity metrics to determine how similar each pair of drugs’ vector space models is to one another. Each similarity value in the medications similarity matrix created during this phase indicates how similar a particular pair of pharmaceuticals is to one another. In data mining, calculating similarity is a frequent task with a large range of potential measures. The Cosine similarity and Euclidean distance are two of the most often used data similarity metrics. It should be emphasized that because the selection of a data similarity metric is domain-specific, it is too challenging to know whether a metric is superior or worse under a general condition.

### 3.1. Computing DDS of Anti-Epileptic Drugs: A Case Study

This section explains how to compute DDS among a specific group of drugs used mostly to treat epilepsy using the methods given above. Anti-Epileptic Drugs (AEDs) are drugs primarily used to treat epilepsy, a neurological condition characterized by a variety of seizure forms, therapeutic sensitivity, and prognosis. Although the currently available AEDs provide greater treatment options for different types of seizures, none of them treats the disease etiology as they all work by suppressing the seizures when they occur. Additionally, more than one-third of epilepsy patients are still unable to manage their seizures using the AEDs that are now available [39].

The AEDs interact with a wide range of various molecular targets to produce their desired effects.The AEDs primarily target two broad target groupings [40]: the specific aspects of the damaged membrane, which are typically regarding aberrant ion permeability (calcium, sodium, and potassium), and the compromised synaptic functioning (heightened excitation or inadequate transmission of suppression). Even though the majority of recently developed AEDs, such as lacosamide and Perampanel, have numerous modes of action, several older AEDs, such as valproate, also have other pharmacological activities that are uncertain in relation to their anticonvulsant activity. Undoubtedly, the ongoing effort to identify the targets of the AEDs that are currently being used will advance knowledge of the pathophysiological mechanisms underlying epileptic seizures and the creation of novel therapeutic approaches.

### 3.2. AEDs Related Patients’ Reviews Extraction

The raw data of AEDs are extracted from Askapatient platform through a web crawler. The extracted data involve patients’ experiences and ratings of AED, reasons for using AED, side effects of AED, comments, gender, age, duration/dosage, and posting dates. When the data have been extracted, the number of AEDs reviews range from 1860 reviews for Lamotrigine to a single review for Aptiom. Therefore, this research does not consider AEDs whose review number in the Askapatient platform is less than 150. Table 1 lists the considered AEDs in this work.

**Figure 2:**
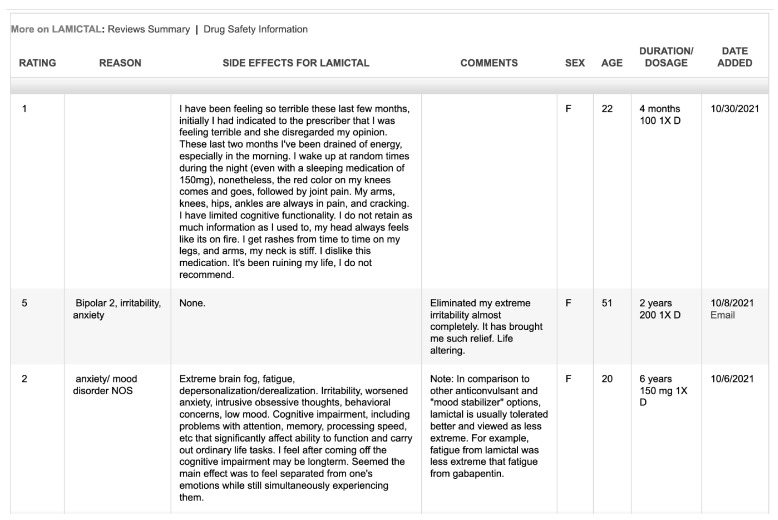
Snapshot of patients’ reviews extracted from Askapatient.com (accessed on 2 January 2023) for Lamictal (Lamotrigine).

Moreover, Figure 2 is a snapshot of the detailed data extracted from Askapatient for Lamictal (Lamotrigine).

### 3.3. AEDs Documents Generation

In this step, the relevant data, which include side effects and comments, for each AED are selected from the extracted patients’ reviews and then compiled into a unified single document for each AED.

### 3.4. AEDs Documents Preprocessing

As pointed out above, some NLP techniques must be applied to preprocess AED documents and transform them into vector space model representation. The applied NLP techniques are

- Text cleaning: eliminating all digits, numerals, and punctuation.
- Normalizing text entails changing capitalization to lowercase.
- Stop words should be eliminated because they have no bearing on the DDS computation.
- Using three as the maximum number of n-grams, all terms in an AED document are used to generate unigrams, bigrams, and trigrams.

### 3.5. Computing DDS of AEDs

In this study, the similarities across AEDs works are determined using four data similarity metrics, including Cosine Similarity, Euclidean Distance, Manhattan Distance, and Jaccard Coefficient, which are widely used in the text similarity area.

#### 3.5.1. Cosine Similarity (CS)

A popular method to gauge text similarity is via the Cosine Similarity (CS) metric [15]. In an inner product space, it calculates the cosine of the angle formed by two non-zero vectors. The vector’s absolute length has no effect on the CS measure. The CS measure between two vectors X = (x1…xn) and Y = (y1…yn) is defined as:

(1) $$CS\left(X,Y\right)=\frac{\sum_{i=1}^{n}x_{i}y_{i}}{\sum_{i=1}^{n}x_{i}^{2}\sum_{i=1}^{n}y_{i}^{2}}$$

An interesting aspect of the CS measure is its variance to linear transformations and invariance to rotation. Additionally, the vector length has no bearing on the CS measure [41].

#### 3.5.2. Euclidian Distance (ED)

The most typical metric employed for geometrical issues is the Euclidean Distance (ED) measure. The straight-line distance in n-dimensional space between any two data points is what is meant by this term. In data mining, it is has been widely applied for many tasks such as clustering problems [42]. Given two vectors representing two data points, X = (x1 …xn) and Y = (y1 …yn), the ED measure between them is defined as follows:

(2) $$ED\left(X,Y\right)=\sqrt{\sum_{i=1}^{n}{\left(x_{i}-y_{i}\right)}^{2}}$$

The ED measure has demonstrated several intriguing qualities, although suffering from a number of issues related to data sparsity, distribution, noise, and feature relevance, particularly in the high-dimensional space. The ED measure’s invariance to rotation, or the fact that the straight-line distance is unaffected by the axis system’s orientation, is an interesting feature [43]. This feature suggests that distance can be applied without being affected by procedures such as singular value decomposition and principal component analysis. The logical interpretability of ED measurements is another essential feature.

#### 3.5.3. Manhattan Distance (MD)

The Manhattan Distance (MD) and ED measures are comparable in that they are both particular instances of the Minkowski distance [43]. In a place such as New York City’s Manhattan island, where the streets are organized into a grid, the MD measure is specified in terms of “city block” distance. Due to its resemblance to the ED measure, MD has the same interesting characteristics of being rotation-invariant and interpretable as the ED measure as well as experiencing the same difficulties in high-dimensional space. The MD measure between two vectors, X = (x1…xn) and Y = (y1…yn), which represent two data points, is defined as:

(3) $$MD\left(X,Y\right)=\sum_{i=1}^{n}\left(x_{i}-y_{i}\right)$$

#### 3.5.4. Jaccard Coefficient (JC)

The Jaccard Coefficient (JC) measure is defined as the similarity between two finite sets by calculating the size of the intersection over the size of the union of the two sets [16]. Thus, if there are no intersecting elements between the two sets, JC equals to zero; however, if all elements intersect, JC equals to one. Given two sets X and Y, the JC measure is defined as follows:

(4) $$JC\left(X,Y\right)=\frac{\left|X\cap Y\right|}{X\cup Y}$$

## 4. Results and Discussion

This section displays the main findings from calculating the degree of similarity between the text documents of AEDs using the four similarity measures. Please see the Table 2, Table 3, Table 4 and Table 5.

The results shown in the above tables indicate that these measures are different as they yield quite different results due to the differences between their working mechanisms. In other words, although these measures evaluate how two documents, represented commonly as two points in the vector space, are related, each measure has a different evaluation of that relationship because what “similarity” means is different for each measure. This is obvious from the differences in their scales and range of similarity values. For example, since Euclidian and Manhattan distance define similarity in terms of the distance between two vectors, their scales fall in the range [0, ∞], where 0 means that the two documents are identical and the more they are dissimilar, the higher the value of these measures. Nonetheless, due to the differences in the meaning of distance between the two measures, Euclidean distance results are somewhat lower than the Manhattan distance measure. More precisely, while Euclidean distance measures the straight distance between two points in the vector space, the Manhattan distance is the sum of absolute differences between points across all the dimensions.

The cosine and Jaccard coefficient measures, on the other hand, deal with the similarity between a two documents from a different perspective. Unlike distance-based similarity measures, these measures interpret the similarity between two documents in terms of the closeness of the two documents to each other; therefore, their scales fall in the range [0, 1], where 0 means the two documents are totally dissimilar and 1 means the two documents are identical. Nonetheless, the two measures are different in their interpretation of the similarity between two documents. While the cosine measure interprets the similarity in terms of the orientation of the two vectors in vector space, the Jaccard coefficient interprets the similarity in terms of the size of the intersection divided by the size of the union of the two sets representing the documents. Another important difference between distance-based measures (Euclidian and Manhattan) and closeness-based measures is that the distance-based measures account for the magnitude of the values representing the dimension, whereas closeness-based measures are much less effected by magnitude, or how large the numbers are.

To overcome the above-mentioned variance in measuring the DDS, a unified scale measurement scale can be used. For this problem, a similarity-based ranking method is applied, where for each drug, the remaining drugs are ordered descendingly based on the obtained DDS from each measure and the ranking values are used instead. The results of applying the similarity-based ranking method are presented in Table 6, Table 7, Table 8 and Table 9.

In contrast to the similarities, the similarity-based rankings look more consistent and illustrate, for each AED, the ranks of the remaining AEDs with respect to their similarity. In addition to the unified measurement scale provided by the similarity-based ranking method, the obtained ranking values allow two types of analyses to be performed. The first analysis is drug-drug correlations which is motivated by the observed consistency between the ranking values of drugs in the rows within each table. The drug-drug correlation analysis would provide insights on the overall drug-drug similarity. The second analysis is the agreement between the similarity measures which is motivated by the observed consistencies between the corresponding drugs ranking values across tables. This analysis would provide insights on the performance of similarity measures relative to each other. For both analyses, rank correlation coefficient methods can be applied. A rank correlation coefficient is used to assess the significance of the relation between two rankings by measuring the degree of similarity between them. In this work, Pearson’s rank correlation coefficient [44] over the obtained drug rankings is defined for two variables X = (x1 …xn) and Y = (y1 …yn) as follows:

(5) $$Pr=\frac{n\sum xy-(\sum x)(\sum y)}{\sqrt{\left[n\sum x^{2}-{\left(\sum x\right)}^{2}\right]\left[n\sum y^{2}-\left(\sum y^{2}\right)\right]}}$$

where Pr is the Pearson correlation coefficient, xi and yi are values of the X and Y variables.

### 4.1. Drug-Drug Correlations Analysis

The drug-drug correlation analysis can be performed by applying Pearson’s rank correlation coefficient to the ranking values of each drugs within the same table. This can be considered as a second-order similarity measuring between AEDs to measure how the drugs are ordered with respect to their similarity to a particular drug. Table 10 presents the degree of agreement between each pair of AEDs in how the other AEDs are ranked measured by each one of the four measures. This unified scale allows to reach a final score of the similarity-based correlations between each pair of AEDs.

Based on the obtained drug-drug similarity-based correlations, an overall AEDs similarity-based correlation can be calculated as shown in Table 11.

### 4.2. Agreement Analysis of Similarity Measures

As pointed out above, the second analysis is the agreement between the similarity measures to provide insights on the performance of similarity measures relative to each other. Again, this analysis is performed by applying the Pearson’s ranked correlation coefficient to the obtained Drug Ranking values presented in Table 6, Table 7, Table 8 and Table 9.

The results of the agreement analysis using Pearson ranked correlation coefficient shows various levels of agreement between the four measures in measuring the similarities between AEDs. The values in the last rows of Table 12 represent the average agreement between different pairs of measures over all AEDs. It is obvious that Euclidian and Manhattan measures have the highest agreement. This can be attributed to the similar working mechanisms of the two measures where they measure the similarity in terms of the distance between the vectors in a Cartesian space. In addition, both Manhattan and Jaccard show a quite high degree of agreement between them, though both measures evaluate the similarity work on a different basis; however, the simplicity of the two measures could interpret the high degree of agreement between them. On the other hand, the Cosine similarity measure shows a low agreement with other measures, where it is the lowest with Jaccard. This reflects the inherent differences of the Cosine measure with others.

### 4.3. Evaluation

**Figure 3:**
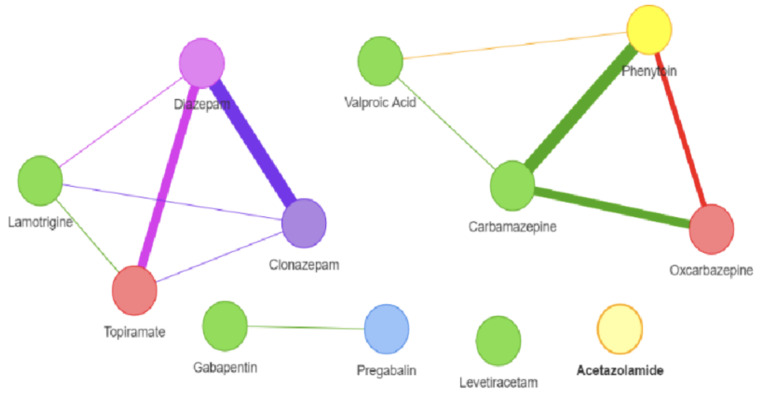
AEDs. Drug-Drug Similarity-based correlations from DrugSimDB.

To evaluate the discovered similarity-based correlations among AEDs, it is meaningful to compare the obtained similarity from social media with the AEDs similarities that are based on drug-centric data mentioned above. For this sake, this research uses the DrugSimDB [45] tool which integrates multiple sources of drug-centric data to compute DDS among a comprehensive list of drugs. It includes 238,635 significant multi-modal DDS for 10,317 small-molecule medications that are either unlawful or withdrawn (2466 approved and 7212 investigational). DrugSimDB uses a variety of public datasets. This covers protein sequences and their functional annotations, drug-induced pathways, chemical structure descriptors, interactions between proteins and proteins and between drugs, to determine the degree to which each combination of medications has the same targets, structures, activities, and routes. DrugSimDB is a web-based application that enables users to browse or download the complete drug database or any crucial processed files. Table 13 presents the results of AEDs similarities obtained from DrugSimDB and Figure 3 shows their representation as a network.

Assuming the average DDS obtained from DrugSimDB tool as ground truth, the evaluation of the AEDs DDS obtained from social media can be performed in terms of Precision, Recall, and F1 as given in the following equations. In doing so, threshold values of AEDs’ drug-drug similarity-based correlations shown in Table 11 need to be specified so as two drugs are considered similar when their similarity-based correlation is above the threshold. Table 14 illustrates the obtained P, R, and F1 values for several threshold values. As shown in Table 14, the best F1 is obtained when the chosen threshold is 0.75. These results provide evidence on the feasibility of using drug-centric data from social media.

## 5. Conclusions

In this research, a framework for computing source data for computing drug-drug similarity based on a novel data source that represents patient perspective on drugs is proposed. The proposed framework employs text similarity methods to compute DDS from patients’ reviews collected from social media. A case study for computing DDS of a specific set of drugs, AEDs, is presented and the obtained results are analyzed using Pearson’s correlation coefficient method to investigate the AEDs DDS and the performance of four similarity measures. The AEDs DDS are compared with DDS obtained from DrugSimDB which depends on the commonly used drug-centric data and the results provide evidence on the feasibility of using drug-centric data for computing DDS. The outcomes of this research are expected to contribute to the healthcare at a practical as well as theoretical level. At the theoretical level, this research is considered the first of its kind to investigate patient’s centric data for computing DDS, which can inspire further research in this direction to fully exploit this novel source of data. At a practical level, this research can inform practical applications of drug discovery and development, which rely on computing DDS, with a new source of data to compensate for missing data across professional data sources and provide a multi-view perspective to compute DDS.

This research can be extended in several directions. First, there are abundant text similarity methods that can be investigated for improving the computation of DDS. Second, more sophisticated NLP methods can be utilized in the preprocessing of the textual data of drug documents to improve the computation of DDS. Finally, for the sake of generality, the proposed DDS framework can be experimented on an extended set of Central Nervous System CNS-acting drugs such as anti-Alzheimer, anti-Parkinson’s, and antipsychotic drugs.

## Figures and Tables

**Table 1:** List of AEDs.

Number	Generic Name	Number of Reviews
1	Carbamazepine	283
2	Oxcarbazepine	357
3	Gabapentin	914
4	Pregabalin	1392
5	Acetazolamide	155
6	Lamotrigine	1845
7	Levetiracetam	190
8	Topiramate	1764
9	Phenytoin	183
10	Diazepam	393
11	Clonazepam	324
12	Klonopin	217
13	Divalproex	783
14	Divalproex-ER	566
	Total	9366

**Table 2:** TAEDs Drug-Drug Similarity using CS Measure.

	Carbamazepine	Oxcarbazepine	Gabapentin	Pregabalin	Acetazolamide	Lamotrigine	Levetiracetam	Topiramate	Phenytoin	Diazepam	Clonazepam	Klonopin	Divalproex	Divalproex-ER
Carbamazepine	1.00	0.65	0.63	0.58	0.45	0.62	0.54	0.58	0.52	0.46	0.54	0.51	0.56	0.56
Oxcarbazepine	0.65	1.00	0.60	0.55	0.47	0.69	0.56	0.59	0.49	0.47	0.57	0.53	0.61	0.62
Gabapentin	0.63	0.60	1.00	0.75	0.46	0.58	0.47	0.56	0.42	0.51	0.56	0.54	0.54	0.54
Pregabalin	0.58	0.55	0.75	1.00	0.44	0.53	0.43	0.53	0.38	0.45	0.49	0.47	0.50	0.51
Acetazolamide	0.45	0.47	0.46	0.44	1.00	0.45	0.38	0.58	0.36	0.34	0.40	0.37	0.42	0.42
Lamotrigine	0.62	0.69	0.58	0.53	0.45	1.00	0.55	0.60	0.48	0.48	0.57	0.55	0.61	0.62
Levetiracetam	0.54	0.56	0.47	0.43	0.38	0.55	1.00	0.47	0.48	0.41	0.48	0.46	0.49	0.49
Topiramate	0.58	0.59	0.56	0.53	0.58	0.60	0.47	1.00	0.43	0.42	0.50	0.47	0.59	0.57
Phenytoin	0.52	0.49	0.42	0.38	0.36	0.48	0.48	0.43	1.00	0.37	0.43	0.41	0.43	0.43
Diazepam	0.46	0.47	0.51	0.45	0.34	0.48	0.41	0.42	0.37	1.00	0.64	0.65	0.41	0.43
Clonazepam	0.54	0.57	0.56	0.49	0.40	0.57	0.48	0.50	0.43	0.64	1.00	0.76	0.49	0.51
Klonopin	0.51	0.53	0.54	0.47	0.37	0.55	0.46	0.47	0.41	0.65	0.76	1.00	0.47	0.49
Divalproex	0.56	0.61	0.54	0.50	0.42	0.61	0.49	0.59	0.43	0.41	0.49	0.47	1.00	0.81
Divalproex-ER	0.56	0.62	0.54	0.51	0.42	0.62	0.49	0.57	0.43	0.43	0.51	0.49	0.81	1.00

**Table 3:** AEDs Drug-Drug Similarity using ED Measure.

	Carbamazepine	Oxcarbazepine	Gabapentin	Pregabalin	Acetazolamide	Lamotrigine	Levetiracetam	Topiramate	Phenytoin	Diazepam	Clonazepam	Klonopin	Divalproex	Divalproex-ER
Carbamazepine	0.00	0.36	0.61	0.97	0.55	0.88	0.46	1.16	0.48	0.59	0.52	0.83	0.57	0.43
Oxcarbazepine	0.36	0.00	0.62	0.98	0.55	0.81	0.45	1.14	0.51	0.60	0.50	0.81	0.52	0.39
Gabapentin	0.61	0.62	0.00	0.59	0.77	0.82	0.76	1.05	0.82	0.72	0.66	0.79	0.70	0.69
Pregabalin	0.97	0.98	0.59	0.00	1.10	1.00	1.12	1.11	1.16	1.07	1.02	1.05	0.98	1.04
Acetazolamide	0.55	0.55	0.77	1.10	0.00	1.04	0.61	1.16	0.63	0.72	0.67	0.98	0.71	0.57
Lamotrigine	0.88	0.81	0.82	1.00	1.04	0.00	0.95	0.94	1.02	0.99	0.88	0.89	0.80	0.90
Levetiracetam	0.46	0.45	0.76	1.12	0.61	0.95	0.00	1.26	0.50	0.65	0.58	0.89	0.65	0.49
Topiramate	1.16	1.14	1.05	1.11	1.16	0.94	1.26	0.00	1.30	1.29	1.19	1.19	1.04	1.18
Phenytoin	0.48	0.51	0.82	1.16	0.63	1.02	0.50	1.30	0.00	0.68	0.63	0.94	0.71	0.55
Diazepam	0.59	0.60	0.72	1.07	0.72	0.99	0.65	1.29	0.68	0.00	0.44	0.65	0.76	0.63
Clonazepam	0.52	0.50	0.66	1.02	0.67	0.88	0.58	1.19	0.63	0.44	0.00	0.52	0.66	0.55
Klonopin	0.83	0.81	0.79	1.05	0.98	0.89	0.89	1.19	0.94	0.65	0.52	0.00	0.88	0.86
Divalproex	0.57	0.52	0.70	0.98	0.71	0.80	0.65	1.04	0.71	0.76	0.66	0.88	0.00	0.35
Divalproex-ER	0.43	0.39	0.69	1.04	0.57	0.90	0.49	1.18	0.55	0.63	0.55	0.86	0.35	0.00

**Table 4:** AEDs Drug-Drug Similarity using MD Measure.

	Carbamazepine	Oxcarbazepine	Gabapentin	Pregabalin	Acetazolamide	Lamotrigine	Levetiracetam	Topiramate	Phenytoin	Diazepam	Clonazepam	Klonopin	Divalproex	Divalproex-ER
Carbamazepine	0.00	7.06	11.47	16.41	9.40	19.22	7.38	21.86	7.72	9.99	9.30	15.27	9.36	7.34
Oxcarbazepine	7.06	0.00	10.51	15.60	9.75	17.65	7.46	21.09	8.89	10.38	9.18	14.79	8.72	7.19
Gabapentin	11.47	10.51	0.00	9.43	14.39	15.06	12.64	17.15	13.70	12.32	10.81	12.86	10.74	12.30
Pregabalin	16.41	15.60	9.43	0.00	18.95	14.79	17.75	15.74	18.75	16.92	15.33	15.01	14.46	17.17
Acetazolamide	9.40	9.75	14.39	18.95	0.00	23.12	9.42	22.97	9.98	12.04	11.96	18.56	12.65	8.95
Lamotrigine	19.22	17.65	15.06	14.79	23.12	0.00	20.29	14.70	21.51	20.37	18.59	16.78	16.19	20.03
Levetiracetam	7.38	7.46	12.64	17.75	9.42	20.29	0.00	23.17	7.54	10.10	9.37	15.62	9.96	7.16
Topiramate	21.86	21.09	17.15	15.74	22.97	14.70	23.17	0.00	24.34	23.18	21.39	19.54	18.85	22.79
Phenytoin	7.72	8.89	13.70	18.75	9.98	21.51	7.54	24.34	0.00	10.82	10.52	16.95	11.41	8.29
Diazepam	9.99	10.38	12.32	16.92	12.04	20.37	10.10	23.18	10.82	0.00	6.89	11.64	11.74	9.98
Clonazepam	9.30	9.18	10.81	15.33	11.96	18.59	9.37	21.39	10.52	6.89	0.00	10.15	10.13	9.30
Klonopin	15.27	14.79	12.86	15.01	18.56	16.78	15.62	19.54	16.95	11.64	10.15	0.00	14.29	15.98
Divalproex	9.36	8.72	10.74	14.46	12.65	16.19	9.96	18.85	11.41	11.74	10.13	14.29	0.00	7.86
Divalproex-ER	7.34	7.19	12.30	17.17	8.95	20.03	7.16	22.79	8.29	9.98	9.30	15.98	7.86	0.00

**Table 5:** AEDs Drug-Drug Similarity using JC Measure.

	Carbamazepine	Oxcarbazepine	Gabapentin	Pregabalin	Acetazolamide	Lamotrigine	Levetiracetam	Topiramate	Phenytoin	Diazepam	Clonazepam	Klonopin	Divalproex	Divalproex-ER
Carbamazepine	1.00	0.51	0.37	0.29	0.40	0.24	0.47	0.25	0.47	0.41	0.44	0.32	0.44	0.45
Oxcarbazepine	0.51	1.00	0.40	0.31	0.39	0.27	0.48	0.27	0.46	0.43	0.47	0.34	0.46	0.46
Gabapentin	0.37	0.40	1.00	0.56	0.25	0.46	0.30	0.48	0.31	0.40	0.42	0.51	0.49	0.27
Pregabalin	0.29	0.31	0.56	1.00	0.20	0.52	0.23	0.53	0.24	0.33	0.34	0.51	0.42	0.20
Acetazolamide	0.40	0.39	0.25	0.20	1.00	0.16	0.40	0.17	0.38	0.32	0.33	0.21	0.30	0.42
Lamotrigine	0.24	0.27	0.46	0.52	0.16	1.00	0.19	0.58	0.21	0.27	0.28	0.48	0.39	0.17
Levetiracetam	0.47	0.48	0.30	0.23	0.40	0.19	1.00	0.20	0.47	0.39	0.42	0.27	0.38	0.48
Topiramate	0.25	0.27	0.48	0.53	0.17	0.58	0.20	1.00	0.21	0.27	0.28	0.48	0.39	0.17
Phenytoin	0.47	0.46	0.31	0.24	0.38	0.21	0.47	0.21	1.00	0.38	0.39	0.27	0.38	0.43
Diazepam	0.41	0.43	0.40	0.33	0.32	0.27	0.39	0.27	0.38	1.00	0.53	0.41	0.41	0.36
Clonazepam	0.44	0.47	0.42	0.34	0.33	0.28	0.42	0.28	0.39	0.53	1.00	0.43	0.46	0.40
Klonopin	0.32	0.34	0.51	0.51	0.21	0.48	0.27	0.48	0.27	0.41	0.43	1.00	0.45	0.23
Divalproex	0.44	0.46	0.49	0.42	0.30	0.39	0.38	0.39	0.38	0.41	0.46	0.45	1.00	0.36
Divalproex-ER	0.45	0.46	0.27	0.20	0.42	0.17	0.48	0.17	0.43	0.36	0.40	0.23	0.36	1.00

**Table 6:** AEDs Drug-Drug Similarity-based Rankings using CS Measure.

	Carbamazepine	Oxcarbazepine	Gabapentin	Pregabalin	Acetazolamide	Lamotrigine	Levetiracetam	Topiramate	Phenytoin	Diazepam	Clonazepam	Klonopin	Divalproex	Divalproex-ER
Carbamazepine	1	2	3	5	14	4	10	6	11	13	9	12	8	7
Oxcarbazepine	3	1	6	10	14	2	9	7	12	13	8	11	5	4
Gabapentin	3	4	1	2	13	5	12	7	14	11	6	9	10	8
Pregabalin	3	4	2	1	12	6	13	5	14	11	9	10	8	7
Acetazolamide	5	3	4	7	1	6	11	2	13	14	10	12	8	9
Lamotrigine	3	2	7	11	14	1	9	6	13	12	8	10	5	4
Levetiracetam	4	2	10	12	14	3	1	9	7	13	8	11	6	5
Topiramate	5	4	8	9	6	2	11	1	13	14	10	12	3	7
Phenytoin	2	3	10	12	14	5	4	6	1	13	7	11	8	9
Diazepam	7	6	4	8	14	5	12	10	13	1	3	2	11	9
Clonazepam	7	5	6	10	14	4	12	9	13	3	1	2	11	8
Klonopin	7	6	5	9	14	4	12	10	13	3	2	1	11	8
Divalproex	6	3	7	8	13	4	10	5	12	14	9	11	1	2
Divalproex-ER	6	3	7	9	14	4	10	5	13	12	8	11	2	1

**Table 7:** AEDs Drug-Drug Similarity-based Rankings using ED Measure.

	Carbamazepine	Oxcarbazepine	Gabapentin	Pregabalin	Acetazolamide	Lamotrigine	Levetiracetam	Topiramate	Phenytoin	Diazepam	Clonazepam	Klonopin	Divalproex	Divalproex-ER
Carbamazepine	1	2	10	13	7	12	4	14	5	9	6	11	8	3
Oxcarbazepine	2	1	10	13	8	12	4	14	6	9	5	11	7	3
Gabapentin	3	4	1	2	10	13	9	14	12	8	5	11	7	6
Pregabalin	3	5	2	1	11	6	13	12	14	10	7	9	4	8
Acetazolamide	2	3	10	13	1	12	5	14	6	9	7	11	8	4
Lamotrigine	6	3	4	12	14	1	10	9	13	11	5	7	2	8
Levetiracetam	3	2	10	13	7	12	1	14	5	8	6	11	9	4
Topiramate	8	6	4	5	7	2	12	1	14	13	11	10	3	9
Phenytoin	2	4	10	13	6	12	3	14	1	8	7	11	9	5
Diazepam	3	4	10	13	9	12	6	14	8	1	2	7	11	5
Clonazepam	4	3	9	13	11	12	7	14	8	2	1	5	10	6
Klonopin	6	5	4	13	12	10	9	14	11	3	2	1	8	7
Divalproex	4	3	7	13	9	11	5	14	8	10	6	12	1	2
Divalproex-ER	4	3	10	13	8	12	5	14	6	9	7	11	2	1

**Table 8:** Drug-Drug Similarity-based Rankings of AEDs using MD Measure.

	Carbamazepine	Oxcarbazepine	Gabapentin	Pregabalin	Acetazolamide	Lamotrigine	Levetiracetam	Topiramate	Phenytoin	Diazepam	Clonazepam	Klonopin	Divalproex	Divalproex-ER
Carbamazepine	1	2	10	12	8	13	4	14	5	9	6	11	7	3
Oxcarbazepine	2	1	10	12	8	13	4	14	6	9	7	11	5	3
Gabapentin	6	3	1	2	12	13	9	14	11	8	5	10	4	7
Pregabalin	9	7	2	1	14	4	12	8	13	10	6	5	3	11
Acetazolamide	3	5	10	12	1	14	4	13	6	8	7	11	9	2
Lamotrigine	9	7	4	3	14	1	11	2	13	12	8	6	5	10
Levetiracetam	3	4	10	12	7	13	1	14	5	9	6	11	8	2
Topiramate	9	7	4	3	11	2	12	1	14	13	8	6	5	10
Phenytoin	3	5	10	12	6	13	2	14	1	8	7	11	9	4
Diazepam	4	6	11	12	10	13	5	14	7	1	2	8	9	3
Clonazepam	4	3	10	12	11	13	6	14	9	2	1	8	7	5
Klonopin	8	6	4	7	13	11	9	14	12	3	2	1	5	10
Divalproex	4	3	7	12	10	13	5	14	8	9	6	11	1	2
Divalproex-ER	4	3	10	12	7	13	2	14	6	9	8	11	5	1

**Table 9:** AEDs Drug-Drug Similarity-based Rankings using JC Measure.

	Carbamazepine	Oxcarbazepine	Gabapentin	Pregabalin	Acetazolamide	Lamotrigine	Levetiracetam	Topiramate	Phenytoin	Diazepam	Clonazepam	Klonopin	Divalproex	Divalproex-ER
carbamazepine	1	2	10	12	9	14	4	13	3	8	7	11	6	5
oxcarbazepine	2	1	9	12	10	14	3	13	7	8	4	11	6	5
gabapentin	10	8	1	2	14	6	12	5	11	9	7	3	4	13
pregabalin	10	9	2	1	14	4	12	3	11	8	7	5	6	13
acetazolamide	4	5	10	12	1	14	3	13	6	8	7	11	9	2
lamotrigine	10	8	5	3	14	1	12	2	11	9	7	4	6	13
levetiracetam	5	3	10	12	7	14	1	13	4	8	6	11	9	2
topiramate	10	9	5	3	14	2	12	1	11	8	7	4	6	13
phenytoin	2	4	10	12	8	14	3	13	1	9	6	11	7	5
Diazepam	6	3	7	11	12	14	8	13	9	1	2	5	4	10
clonazepam	5	3	7	11	12	14	8	13	10	2	1	6	4	9
Klonopin	10	9	2	3	14	4	12	5	11	8	7	1	6	13
divalproex	6	3	2	7	14	10	12	9	11	8	4	5	1	13
divalproex-ER	4	3	10	12	6	14	2	13	5	8	7	11	9	1

**Table 10:** AEDs Drug-Drug Similarity-based Correlations.

		Carbamazepine	Oxcarbazepine	Gabapentin	Pregabalin	Acetazolamide	Lamotrigine	Levetiracetam	Topiramate	Phenytoin	Diazepam	Clonazepam	Klonopin	Divalproex	Divalproex-ER
Carbamazepine	CS	1.00	0.98	0.31	−0.48	0.84	−0.61	0.96	−0.64	0.90	0.72	0.72	0.05	0.85	0.94
	ED	1.00	0.99	0.33	−0.07	0.91	0.03	0.96	−0.50	0.93	0.71	0.63	0.28	0.81	0.88
	MD	1.00	0.98	0.31	−0.48	0.84	−0.61	0.96	−0.64	0.90	0.72	0.72	0.05	0.85	0.94
	JC	1.00	0.93	−0.56	−0.69	0.76	−0.71	0.89	−0.74	0.97	0.48	0.52	−0.66	0.05	0.89
Oxcarbazepine	CS	0.85	1.00	0.62	0.62	0.39	0.98	0.74	0.69	0.53	0.15	0.27	0.22	0.89	0.90
	ED	0.99	1.00	0.37	−0.02	0.87	0.13	0.96	−0.46	0.89	0.72	0.67	0.34	0.86	0.91
	MD	0.98	1.00	0.36	−0.41	0.81	−0.56	0.93	−0.58	0.85	0.67	0.71	0.07	0.90	0.96
	JC	0.93	1.00	−0.45	−0.58	0.71	−0.62	0.87	−0.64	0.88	0.61	0.68	−0.56	0.20	0.86
Gabapentin	CS	0.86	0.62	1.00	0.95	0.45	0.57	0.12	0.38	0.05	0.48	0.45	0.47	0.49	0.50
	ED	0.33	0.37	1.00	0.78	0.24	0.21	0.26	0.02	0.15	0.30	0.33	0.36	0.44	0.31
	MD	0.31	0.36	1.00	0.54	0.07	0.15	0.21	0.11	0.08	0.23	0.41	0.61	0.55	0.27
	JC	−0.56	−0.45	1.00	0.96	−0.85	0.88	−0.74	0.88	−0.64	0.09	0.06	0.96	0.73	−0.77
Pregabalin	CS	0.87	0.62	0.95	1.00	0.56	0.57	0.06	0.53	0.00	0.30	0.25	0.27	0.59	0.58
	ED	−0.07	−0.02	0.78	1.00	−0.14	0.55	−0.20	0.50	−0.29	−0.11	−0.02	0.21	0.20	0.02
	MD	−0.48	−0.41	0.54	1.00	−0.74	0.85	−0.59	0.80	−0.71	−0.43	−0.22	0.51	−0.13	−0.51
	JC	−0.69	−0.58	0.96	1.00	−0.93	0.94	−0.83	0.95	−0.75	−0.07	−0.10	0.95	0.57	−0.86
Acetazolamide	CS	0.51	0.39	0.45	0.56	1.00	0.36	0.01	0.80	−0.01	−0.27	−0.22	−0.26	0.41	0.33
	ED	0.91	0.87	0.24	−0.14	1.00	−0.14	0.87	−0.42	0.87	0.59	0.47	0.14	0.70	0.78
	MD	0.84	0.81	0.07	−0.74	1.00	−0.84	0.89	−0.77	0.87	0.62	0.53	−0.17	0.63	0.85
	JC	0.76	0.71	−0.85	−0.93	1.00	−0.97	0.89	−0.98	0.79	0.18	0.24	−0.90	−0.39	0.93
Lamotrigine	CS	0.79	0.98	0.57	0.57	0.36	1.00	0.72	0.71	0.49	0.20	0.34	0.29	0.87	0.90
	ED	0.03	0.13	0.21	0.55	−0.14	1.00	−0.05	0.49	−0.20	−0.01	0.15	0.42	0.39	0.19
	MD	−0.61	−0.56	0.15	0.85	−0.84	1.00	−0.70	0.97	−0.82	−0.67	−0.49	0.10	−0.35	−0.63
	JC	−0.71	−0.62	0.88	0.94	−0.97	1.00	−0.86	0.99	−0.78	−0.16	−0.18	0.92	0.49	−0.89
Levetiracetam	CS	0.51	0.74	0.12	0.06	0.01	0.72	1.00	0.34	0.82	−0.16	−0.01	−0.07	0.58	0.58
	ED	0.96	0.96	0.26	−0.20	0.87	−0.05	1.00	−0.59	0.94	0.72	0.64	0.27	0.75	0.82
	MD	0.96	0.93	0.21	−0.59	0.89	−0.70	1.00	−0.73	0.94	0.72	0.67	−0.03	0.79	0.96
	JC	0.89	0.87	−0.74	−0.83	0.89	−0.86	1.00	−0.88	0.92	0.34	0.38	−0.80	−0.24	0.99
Topiramate	CS	0.59	0.69	0.38	0.53	0.80	0.71	0.34	1.00	0.22	−0.24	−0.12	−0.18	0.77	0.71
	ED	−0.50	−0.46	0.02	0.50	−0.42	0.49	−0.59	1.00	−0.66	−0.73	−0.68	−0.40	−0.16	−0.33
	MD	−0.64	−0.58	0.11	0.80	−0.77	0.97	−0.73	1.00	−0.85	−0.75	−0.56	0.02	−0.39	−0.65
	JC	−0.74	−0.64	0.88	0.95	−0.98	0.99	−0.88	1.00	−0.79	−0.14	−0.17	0.92	0.47	−0.91
Phenytoin	CS	0.46	0.53	0.05	0.00	−0.01	0.49	0.82	0.22	1.00	−0.23	−0.09	−0.16	0.33	0.30
	ED	0.93	0.89	0.15	−0.29	0.87	−0.20	0.94	−0.66	1.00	0.64	0.55	0.16	0.66	0.77
	MD	0.90	0.85	0.08	−0.71	0.87	−0.82	0.94	−0.85	1.00	0.67	0.57	−0.13	0.65	0.87
	JC	0.97	0.88	−0.64	−0.75	0.79	−0.78	0.92	−0.79	1.00	0.38	0.42	−0.72	−0.09	0.89
Diazepam	CS	0.16	0.15	0.48	0.30	−0.27	0.20	−0.16	−0.24	−0.23	1.00	0.96	0.98	−0.07	0.07
	ED	0.71	0.72	0.30	−0.11	0.59	−0.01	0.72	−0.73	0.64	1.00	0.96	0.71	0.46	0.54
	MD	0.72	0.67	0.23	−0.43	0.62	−0.67	0.72	−0.75	0.67	1.00	0.94	0.42	0.60	0.65
	JC	0.48	0.61	0.09	−0.07	0.18	−0.16	0.34	−0.14	0.38	1.00	0.99	0.05	0.63	0.30
Clonazepam	CS	0.19	0.27	0.45	0.25	−0.22	0.34	−0.01	−0.12	−0.09	0.96	1.00	0.99	0.05	0.19
	ED	0.63	0.67	0.33	−0.02	0.47	0.15	0.64	−0.68	0.55	0.96	1.00	0.84	0.45	0.50
	MD	0.72	0.71	0.41	−0.22	0.53	−0.49	0.67	−0.56	0.57	0.94	1.00	0.58	0.69	0.63
	JC	0.52	0.68	0.06	−0.10	0.24	−0.18	0.38	−0.17	0.42	0.99	1.00	0.01	0.64	0.35
Klonopin	CS	0.17	0.22	0.47	0.27	−0.26	0.29	−0.07	−0.18	−0.16	0.98	0.99	1.00	0.01	0.14
	ED	0.28	0.34	0.36	0.21	0.14	0.42	0.27	−0.40	0.16	0.71	0.84	1.00	0.31	0.26
	MD	0.05	0.07	0.61	0.51	−0.17	0.10	−0.03	0.02	−0.13	0.42	0.58	1.00	0.26	−0.02
	JC	−0.66	−0.56	0.96	0.95	−0.90	0.92	−0.80	0.92	−0.72	0.05	0.01	1.00	0.63	−0.83
Divalproex	CS	0.71	0.89	0.49	0.59	0.41	0.87	0.58	0.77	0.33	−0.07	0.05	0.01	1.00	0.98
	ED	0.81	0.86	0.44	0.20	0.70	0.39	0.75	−0.16	0.66	0.46	0.45	0.31	1.00	0.96
	MD	0.85	0.90	0.55	−0.13	0.63	−0.35	0.79	−0.39	0.65	0.60	0.69	0.26	1.00	0.89
	JC	0.05	0.20	0.73	0.57	−0.39	0.49	−0.24	0.47	−0.09	0.63	0.64	0.63	1.00	−0.27
Divalproex-ER	CS	0.70	0.90	0.50	0.58	0.33	0.90	0.58	0.71	0.30	0.07	0.19	0.14	0.98	1.00
	ED	0.88	0.91	0.31	0.02	0.78	0.19	0.82	−0.33	0.77	0.54	0.50	0.26	0.96	1.00
	MD	0.94	0.96	0.27	−0.51	0.85	−0.63	0.96	−0.65	0.87	0.65	0.63	−0.02	0.89	1.00
	JC	0.89	0.86	−0.77	−0.86	0.93	−0.89	0.99	−0.91	0.89	0.30	0.35	−0.83	−0.27	1.00

**Table 11:** Average AEDs Drug-Drug Similarity-based Correlations.

AVG	Carbamazepine	Oxcarbazepine	Gabapentin	Pregabalin	Acetazolamide	Lamotrigine	Levetiracetam	Topiramate	Phenytoin	Diazepam	Clonazepam	Divalproex	Divalproex-ER
Carbamazepine	1.00	0.94	0.23	−0.09	0.75	−0.13	0.83	−0.32	0.82	0.52	0.52	0.60	0.85
Oxcarbazepine	0.94	1.00	0.22	−0.10	0.70	−0.01	0.87	−0.25	0.78	0.54	0.58	0.71	0.91
Gabapentin	0.23	0.22	1.00	0.81	−0.02	0.45	−0.04	0.35	−0.09	0.27	0.31	0.55	0.08
Pregabalin	−0.09	−0.10	0.81	1.00	−0.31	0.73	−0.39	0.69	−0.44	−0.08	−0.02	0.31	−0.19
Acetazolamide	0.75	0.70	−0.02	−0.31	1.00	−0.40	0.66	−0.34	0.63	0.28	0.25	0.34	0.72
Lamotrigine	−0.13	−0.01	0.45	0.73	−0.40	1.00	−0.22	0.79	−0.33	−0.16	−0.05	0.35	−0.11
Levetiracetam	0.83	0.87	−0.04	−0.39	0.66	−0.22	1.00	−0.47	0.90	0.40	0.42	0.47	0.84
Topiramate	−0.32	−0.25	0.35	0.69	−0.34	0.79	−0.47	1.00	−0.52	−0.47	−0.38	0.17	−0.30
Phenytoin	0.82	0.78	−0.09	−0.44	0.63	−0.33	0.90	−0.52	1.00	0.37	0.36	0.39	0.71
Diazepam	0.52	0.54	0.27	−0.08	0.28	−0.16	0.40	−0.47	0.37	1.00	0.96	0.41	0.39
Clonazepam	0.52	0.58	0.31	−0.02	0.25	−0.05	0.42	−0.38	0.36	0.96	1.00	0.46	0.42
Divalproex	0.60	0.71	0.55	0.31	0.34	0.35	0.47	0.17	0.39	0.41	0.46	1.00	0.64
Divalproex-ER	0.85	0.91	0.08	−0.19	0.72	−0.11	0.84	−0.30	0.71	0.39	0.42	0.64	1.00

**Table 12:** AEDs Drug-Drug Similarities from DrugSimDB.

	CS & ED	ED & MD	CS & MD	CS & JC	ED & JC	MD & JC	AVG. Performance
Carbamazepine	0.11	0.99	0.13	0.10	0.95	0.97	0.54
Oxcarbazepine	0.34	0.98	0.33	0.31	0.96	0.95	0.65
Gabapentin	0.64	0.94	0.58	0.60	0.24	0.40	0.57
Pregabalin	0.84	0.78	0.69	0.62	0.52	0.89	0.72
Acetazolamide	0.11	0.96	0.02	−0.01	0.95	1.00	0.51
Lamotrigine	0.84	0.60	0.55	0.32	0.48	0.93	0.62
Levetiracetam	0.56	0.97	0.53	0.46	0.96	0.98	0.74
Topiramate	0.87	0.89	0.65	0.30	0.61	0.86	0.70
Phenytoin	0.51	0.99	0.45	0.56	0.96	0.96	0.74
Diazepam	0.40	0.96	0.27	0.53	0.74	0.71	0.60
Clonazepam	0.57	0.95	0.42	0.51	0.85	0.89	0.70
Klonopin	0.78	0.86	0.73	0.58	0.22	0.49	0.61
Divalproex	0.47	0.98	0.42	0.34	0.23	0.29	0.46
Divalproex-ER	0.35	0.95	0.20	0.02	0.85	0.95	0.55
Average Performance	0.53	0.91	0.43	0.37	0.68	0.80	

**Table 13:** Agreement Analysis of Similarity Measures Performance.

Drug_1	Drug_2	Structure Similarity	Target Similarity	Pathway Similarity	GO_CC Similarity	GO_MF Similarity	GO_BP Similarity	Average
Clonazepam	Diazepam	0.47	0.95	1	0.85	0.86	0.85	0.796
Carbamazepine	Phenytoin	0.42	0.65	NA	0.92	0.95	0.9	0.768
Carbamazepine	Oxcarbazepine	0.64	0.48	NA	0.77	0.8	0.78	0.694
Oxcarbazepine	Phenytoin	0.38	0.57	NA	0.79	0.79	0.83	0.672
Diazepam	Topiramate	0	0.86	1	0.84	0.8	0.72	0.644
Diazepam	Lamotrigine	0.22	0.78	NA	0.69	0.75	0.7	0.628
Clonazepam	Lamotrigine	0.2	0.74	NA	0.72	0.7	0.68	0.608
Lamotrigine	Topiramate	0	0.73	NA	0.65	0.82	0.75	0.59
Clonazepam	Topiramate	0	0.83	1	0.64	0.73	0.66	0.572
Phenytoin	Valproic Acid	0	0.57	NA	0.65	0.73	0.61	0.512
Gabapentin	Pregabalin	0.21	0.34	1	0.69	0.67	0.62	0.506
Carbamazepine	Valproic Acid	0.01	0.41	NA	0.74	0.72	0.6	0.496

**Table 14:** AEDs Drug-Drug Similarity Evaluation Results.

Threshold	0.5	0.6	0.7	0.75	0.80
Precision (P)	0.29	0.35	0.44	0.54	0.40
Recall (R)	0.67	0.67	0.67	0.58	0.33
F1	0.40	0.46	0.53	0.56	0.36

## Data Availability

Not applicable.
